# Elucidating the Molecular Mechanism of CO_2_ Capture by Amino Acid Ionic Liquids

**DOI:** 10.1021/jacs.3c03613

**Published:** 2023-07-13

**Authors:** Bohak Yoon, Gregory A. Voth

**Affiliations:** Department of Chemistry, Chicago Center for Theoretical Chemistry, James Franck Institute, and Institute for Biophysical Dynamics, The University of Chicago, Chicago, Illinois 60637, United States

## Abstract

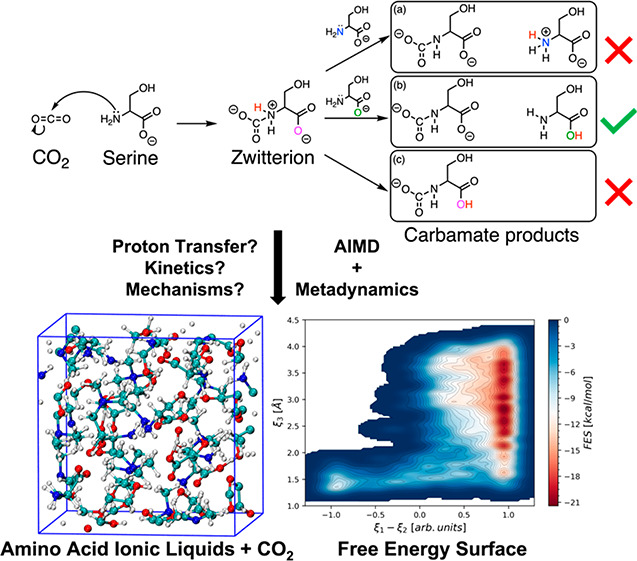

Amino acid ionic liquids have received increasing attention as ideal candidates for the CO_2_ chemisorption process. However, the underlying molecular mechanisms, especially those involving proton transfer, remain unclear. In this work, we elucidate the atomistic-level reaction mechanisms responsible for carbamate formation during CO_2_ capture by amino acid ionic liquids through explicit *ab initio* molecular dynamics augmented by well-tempered metadynamics. The resulting *ab initio* free-energy sampling reveals a two-step reaction pathway in which a zwitterion, initially formed from the reaction between the anion of serine and CO_2_, undergoes a kinetically facile intermolecular proton transfer to the O atom of the COO^–^ moiety in the nearby serine. Further analysis reveals that the significantly reduced free-energy barriers are attributed to enhanced intermolecular interaction between the zwitterion and serine, thus facilitating the kinetic favorability of the proton transfer, which governs the overall CO_2_ capture mechanism. This work provides valuable insight into the important mechanistic and kinetic features of these reactions from explicit condensed phase *ab initio* MD free-energy sampling of the CO_2_ capture process.

The increase of carbon dioxide (CO_2_) emissions into the atmosphere due to anthropogenic activities is a major cause of global warming.^1,2^ As about 80% of the total CO_2_ emissions are attributed to fossil fuel consumption, substantial efforts are urgently needed to mitigate CO_2_ release into the environment.^3,4^ Chemical absorption with aqueous amine solvents currently remains as the most widely implemented technology for post-combustion CO_2_ capture.^5^ However, the extensive implementation of this approach suffers from several drawbacks, including thermal and oxidative degradation of the amine solvents^6,7^ and corrosion^8^ that lead to costly replacement of the equipment and require high energy utilization for the reclamation process.^9,10^ In addition, the makeup of solvent losses constitutes about 20% of the total cost; degradation products can be hazardous if exposed to the environment.^9−12^ As greener alternatives, ionic liquids (ILs) appear to be promising candidates for post-combustion CO_2_ capture through the chemisorption process due to their low vapor pressure, large chemical tunability, and high thermal stability.^13−16^ Among various types of ILs, amino acid ionic liquids (AAILs) can be considered as advantageous over conventional ILs due to their biocompatibility and cost-effective synthesis.^17,18^ AAILs are known to undergo reactions to form carbamate products through a zwitterion intermediate,^19^ similar to aqueous amine solvents.^20^ A few theoretical studies based on density functional theory (DFT) calculations have attempted to investigate the reaction mechanisms for CO_2_ chemisorption by AAILs.^21,22^ However, the molecular mechanism of CO_2_ capture remains unclear and cannot be solely explained by static DFT calculations involving a few molecules in the gas phase or an implicit solvent model. Since the chemisorption process involving proton transfer is dynamic, it requires a careful fundamental understanding of its dynamic and kinetic aspects in condensed phase ILs. In this work, we investigate the molecular mechanisms of CO_2_ chemisorption by AAILs through fully explicit *ab initio* molecular dynamics and well-tempered metadynamics^23,24^ free energy sampling. We thoroughly analyze the free-energy barriers and, thus, kinetic favorability for various possible reaction routes from the zwitterion intermediate involving dynamic proton transfer that leads to carbamate formation. Subsequently, we further assess key dynamical aspects of the proton transfer by investigating the nature of intermolecular interactions that may largely govern the CO_2_ capture process.

*Ab initio* molecular dynamics (AIMD) simulations based on density functional theory were performed with the CP2K software.^25^ To prepare the AIMD simulations, all-atom classical molecular dynamics simulations were first computed using the LAMMPS MD software.^26^ The simulation box contained 15 amino acid ionic liquid ion pairs consisting of choline cations and serine anions with five CO_2_ molecules, while the system was prepared by using PACKMOL.^27^ The cubic simulation box was run for 10 ns in the constant NPT ensemble from which the equilibrated density is obtained. Thereafter, the simulation box was equilibrated under the NVT ensemble for 5 ns, followed by production runs for 15 ns. The final configuration from the production runs is used as the initial structure for the AIMD runs. All AIMD simulations are computed at 300 K. A more detailed description of AIMD simulations can be found in the Supporting Information. Thereafter, well-tempered metadynamics simulations^23,24^ in conjunction with the PLUMED plug-in^28^ were carried out for the free-energy sampling of reaction pathways. More details on the metadynamics simulation setup, convergence tests, and transition state verifications can be found in the Supporting Information.

Chemisorption of CO_2_ by AAILs can be initiated from nucleophilic attack by a basic N atom in serine (anion) at an electrophilic C atom in CO_2_, forming a zwitterion. Thereafter, the zwitterion may undergo three possible routes involving proton transfer for its conversion to carbamate products. The overall reaction pathways are depicted in Figure 1. Here, the three possible routes from the zwitterion intermediate include the following: (a) intermolecular proton transfer to the O atom in serine, (b) intermolecular proton transfer to the N atom in serine, and (c) intramolecular proton transfer to the O atom within the zwitterion (as shown in Figure 1). To investigate the thermodynamic and kinetic favorability of the reaction paths described herein, we first carried out *ab initio* metadynamics simulations. A representative simulation setup with 15 AAIL pairs of cholinium (cation) and serine (anion) with five CO_2_ molecules in a cubic periodic box is illustrated in Figure 2.

**Figure 1:**
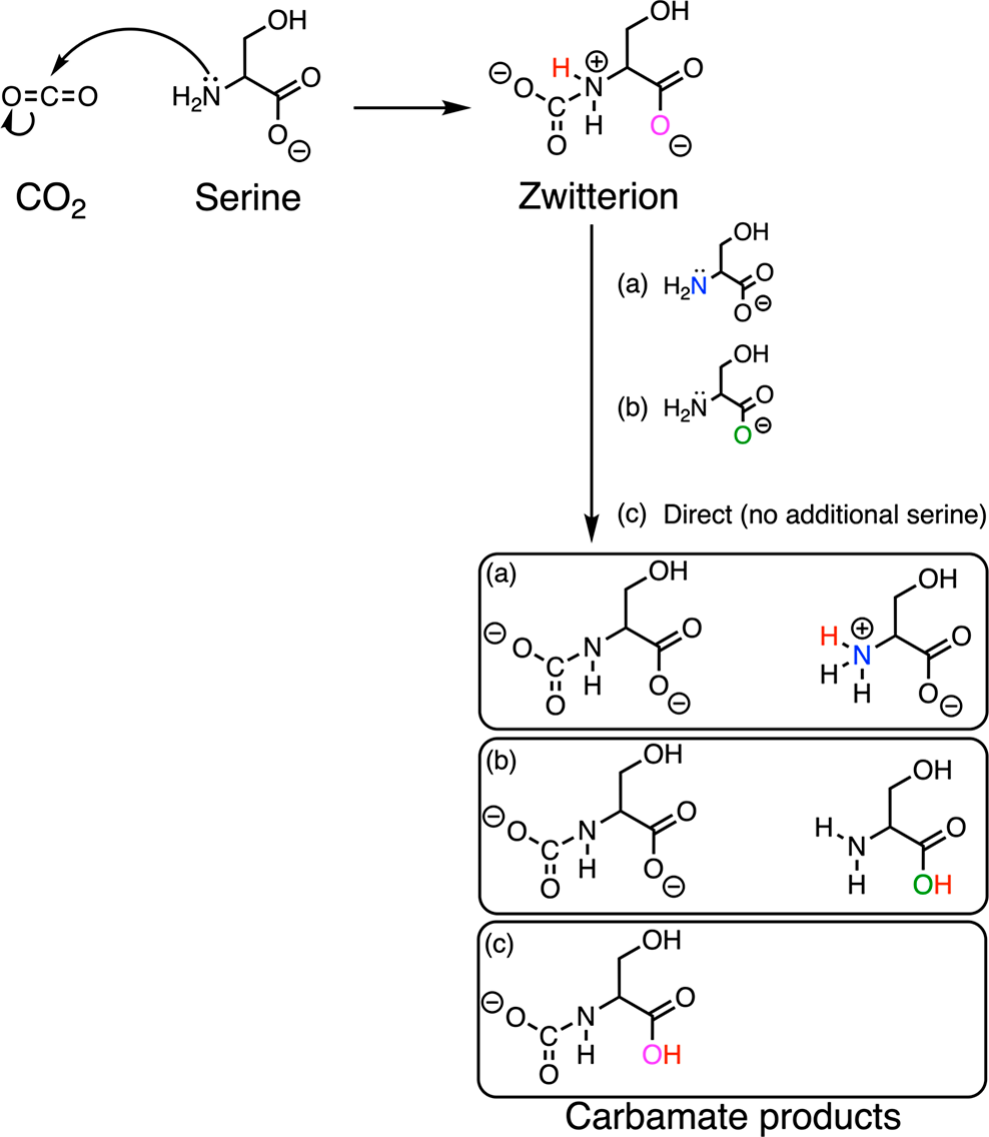
Two-step reaction pathways for CO_2_ capture by amino acid ionic liquids (AAILs) investigated in this work. The first reaction route involves an anion of amino acid ionic liquid (i.e., serine) reacting with CO_2_ to form zwitterion. Thereafter, the second step involves zwitterion converting to carbamate products via proton transfer (a) intermolecularly to a nitrogen atom (N in blue) in serine, (b) intermolecularly to an oxygen atom (O in green) in serine, and (c) intramolecularly to an oxygen atom (O in pink) within zwitterion.

**Figure 2:**
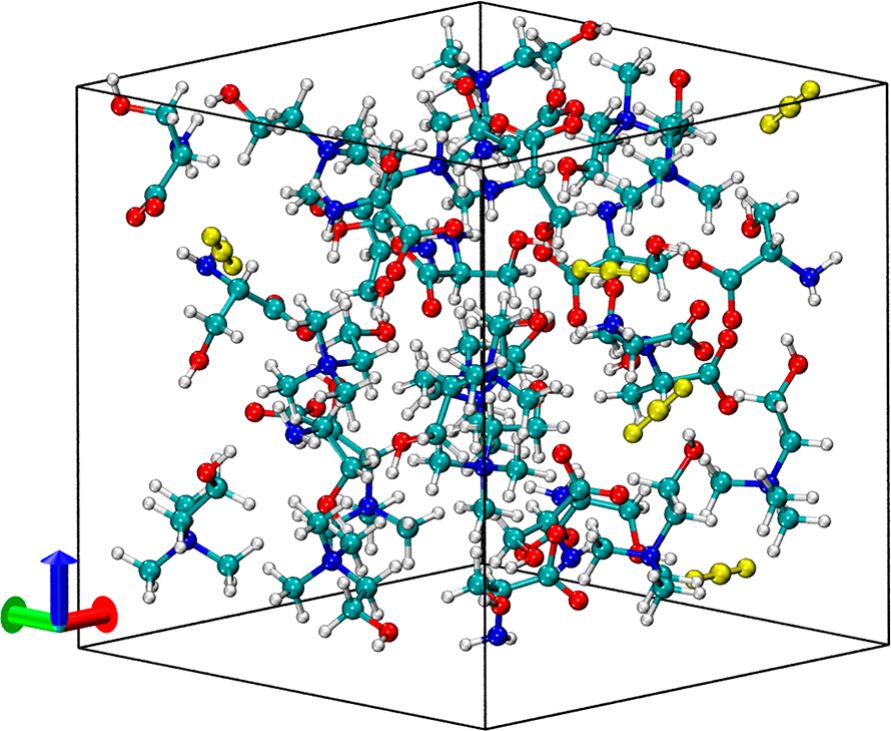
Representative snapshot of a cubic simulation box with periodic boundary conditions containing 15 amino acid ionic liquids with cation and anion pairs of cholinium and serine molecules with five CO_2_ molecules (highlighted in yellow) with a side length of 17.42 Å.

Two-dimensional free-energy surfaces (FES) for the chemisorption of CO_2_ by the AAILs were computed from the AIMD with well-tempered metadynamics. Here, the two-step reaction mechanisms are described by two collective variables (CVs). A first step involving the zwitterion formation from the initial reaction between serine and CO_2_ is described by a single CV of ξ_3_, a bond distance between the N atom in serine and the C atom in CO_2_, illustrating a nucleophilic attack by serine to CO_2_. A relatively larger ξ_3_ denotes a separation between two molecules with no significant interaction, while a relatively smaller ξ_3_ describes bond formation for zwitterion formation. The reaction between serine (anion) and CO_2_ is solely considered, as cholinium (cation) is not directly involved. A second step involving proton transfer from the zwitterion to three carbamate products [(a), (b), and (c)] is described by a CV of ξ_1_-ξ_2_, a linear combination of two coordination numbers representing protonation states. Here, ξ_1_ represents coordination numbers between the N and H atoms in the zwitterion, while ξ_2_ denotes coordination numbers between the N or the O atom in nearby serine and the H atom in the zwitterion. Here, we note that each coordination number only takes into consideration N–H or O–H bonds formed/broken once the proton is transferred from/to, and its value ranges from 0 to 1 for convenience. As coordination numbers are bond distance-dependent, an average bond length of N–H (O–H) is chosen to be 1.1 (1.2) Å, obtained from the first peak position of radial distribution functions with three independent AIMD runs under the canonical ensemble. A distance larger than this average distance guarantees that bonds are broken. ξ_1_ (ξ_2_) has a value close to one if the proton is bound to the zwitterion (serine) and a value close to zero if the proton is released. Thus, a linear combination (ξ_1_-ξ_2_), ranging from −1 to 1, of these two coordination numbers conveniently denotes protonation states of both the zwitterion and nearby serine molecules. For instance, a positive value of CV (ξ_1_-ξ_2_) indicates a state prior to proton transfer from the zwitterion, while a negative value of CV denotes a state with a successful proton transfer from the zwitterion to form carbamate products. A more detailed description of the CVs can be found in the Supporting Information.

Figure 3 shows the FES for two-step reaction paths starting from serine and CO_2_ with three possible proton transfers from the zwitterion: (a) intermolecular transfer to the N atom in nearby serine, (b) intermolecular transfer to the O atom in nearby serine, and (c) intramolecular transfer to the O atom within the zwitterion. The predicted free-energy barriers from AIMD-metadynamics simulations are 17.2 ± 0.2, 12.1 ± 0.2, and 22.5 ± 0.3 kcal/mol for the (a), (b), and (c) cases, respectively, which are reasonably compatible with both experimental and theoretical findings on the predicted barriers for carbamate formation by aqueous amine solvents.^29^ Our FES results reveal that the intermolecular proton transfer to the O atom in serine is most kinetically facile (with the lowest free-energy barrier), followed by the intermolecular proton transfer to the N site and by the intramolecular proton transfer to the O site. Thus, carbamate products through the intermolecular proton transfer to the O atom in serine are expected to be the most dominant. It is important to note that our AIMD-metadynamics can capture the important dynamics of explicit molecules with a quantum mechanical (QM) accuracy. Most importantly, our condensed phase free-energy sampling considers reaction mechanisms and kinetics, including configurational and conformational entropy contributions, which cannot be described with static (e.g., minimum energy) QM calculations.^21,22^

**Figure 3:**
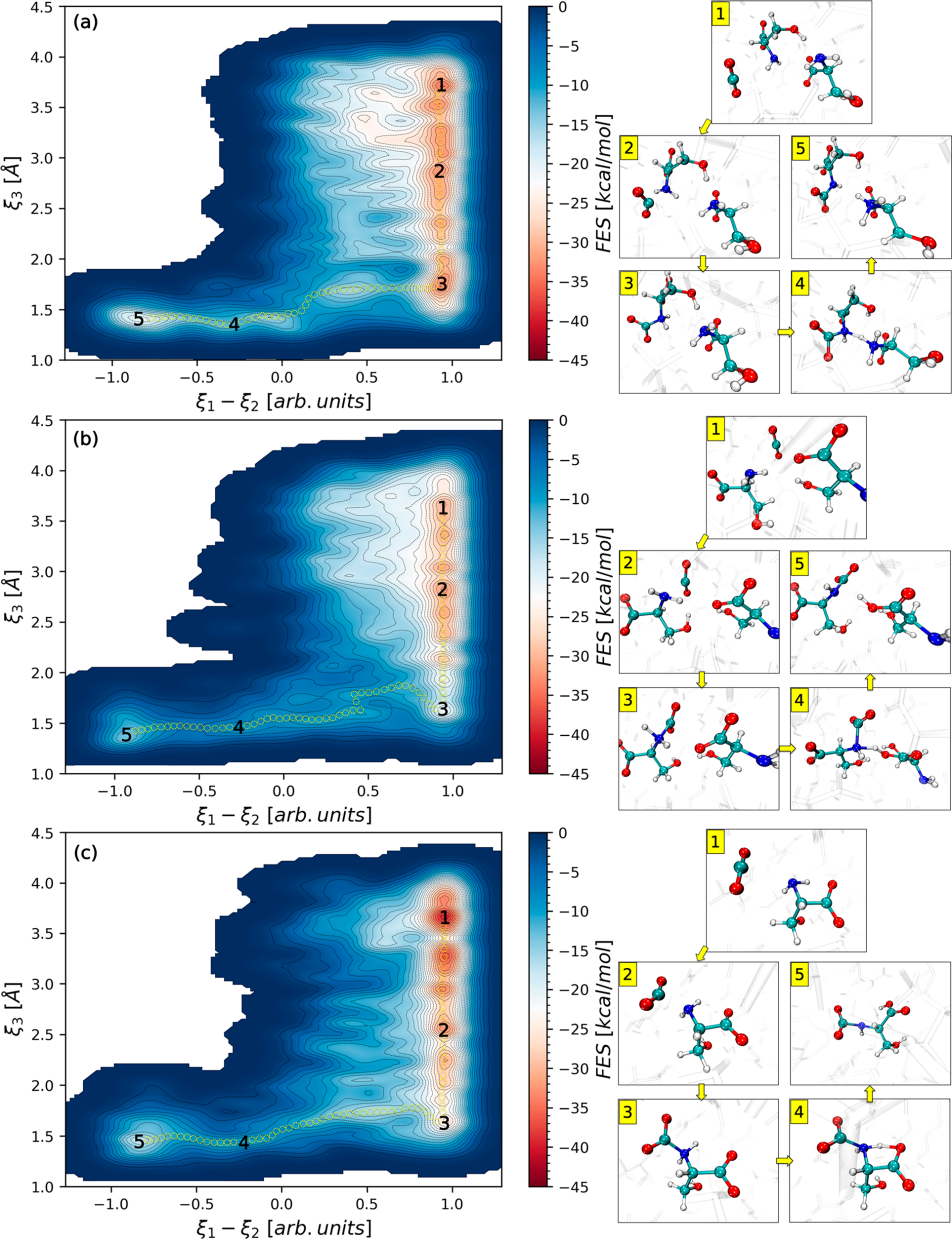
Free-energy surfaces, FES (in kcal/mol), for carbamate formation from the reaction between serine and CO_2_ through zwitterion formation computed from AIMD-metadynamics. The three reaction routes describe zwitterion undergoing proton transfer (a) intermolecularly to the N atom, (b) intermolecularly to the O atom, and (c) intramolecularly within the zwitterion. The reaction path (1 → 2 → 3 → 4 → 5) following the minimum energy pathway (in yellow circle) in each case is shown in FES on the left, while the corresponding molecular configurations for the three cases are shown on the right. The cyan, white, red, and blue balls represent C, H, O, and N atoms, respectively.

To further investigate the primary reasons for the kinetic favorability of proton transfer from the zwitterion, the radial distribution function (RDF), *g*_zwitterion-serine_(*r*), between the zwitterion and serine is computed from AIMD simulations. Here, *g*_zwitterion-serine_(*r*) describes features of intermolecular interactions between a proton in the zwitterion (*H*_zwitterion_) and the N atom (*N*_serine_) or the O atom (*O*_serine_) in serine. As shown in Figure 4, a sharper first-peak position of *H*_zwitterion_-*O*_serine_ at a much shorter distance, *r*, at 2 Å implies a stronger interaction of the proton in the zwitterion with the O site of the COO^–^ moiety in serine than with the N site in serine, which has a broader first-peak of *H*_zwitterion_-*N*_serine_ at a larger *r* of 3.3 Å. The enhanced intermolecular interaction between the zwitterion and the O atom in serine reveals a key reason for the kinetically more facile proton transfer from the zwitterion. This is consistent with our *ab initio* free-energy sampling results in Figure 3 that the barrier height for intermolecular proton transfer from the zwitterion to the O atom in serine is also considerably lower than that to the N atom in serine (or the lowest among three reaction pathways), thereby depicting its enhanced kinetic preference facilitating carbamate formation. Our RDF analysis clearly illustrates that CO_2_ capture by AAILs can be governed by the dynamic features of intermolecular interactions and consequential kinetic favorability, which again cannot be explained with static QM calculations alone.^21,22^

**Figure 4:**
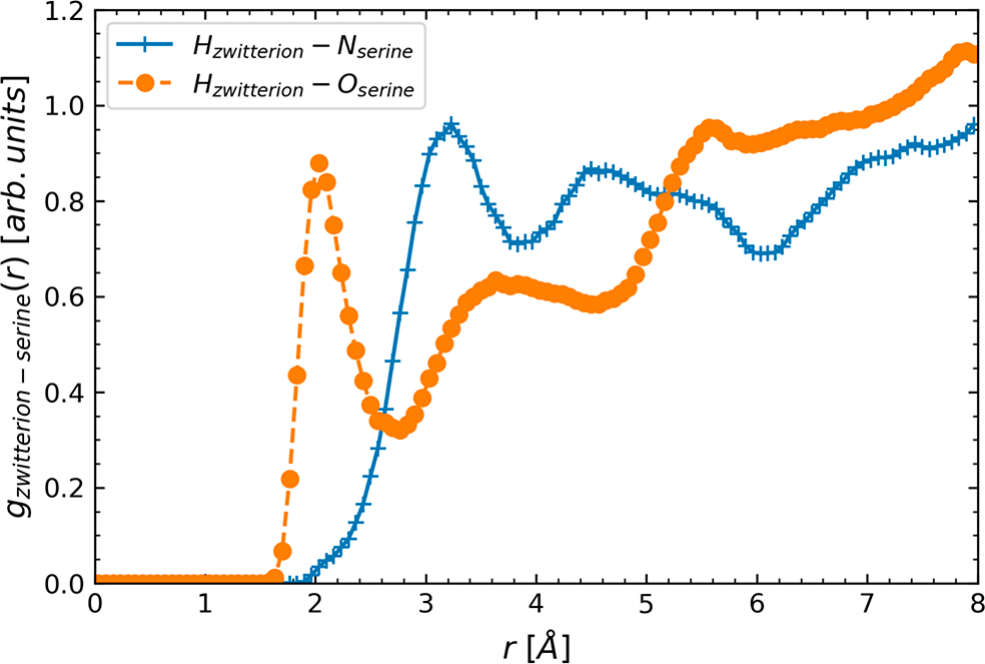
Radial distribution function, *g*_zwitterion-serine_(*r*), between a proton in zwitterion (*H*_zwitterion_) and the N atom in serine, *N*_serine_ (in blue solid line with + marks), or the O atom in serine, *O*_serine_ (in orange dotted line with circular marks), from AIMD simulations.

Our work elucidates the molecular mechanisms of CO_2_ capture by amino acid ionic liquids (AAILs) through a facile proton transfer mechanism. Two-step reaction pathways leading to carbamate formation from the reaction between CO_2_ and serine are investigated, from which a zwitterion, first formed from the initial reaction, undergoes inter- and intramolecular proton transfer. Using *ab initio* molecular dynamics augmented with well-tempered metadynamics, we analyze both the thermodynamic and kinetic favorability of three possible routes involving the proton transfer from the zwitterion during the CO_2_ chemisorption process by AAILs. This is the first time that fully explicit condensed phase free-energy sampling has been carried out to characterize the reaction mechanisms and dynamic features that largely govern CO_2_ capture by AAILs. Further analysis of radial distribution functions reveals that proton transfer to the O site of the COO^–^ moiety in nearby serine can be the most kinetically facile pathway from the zwitterion due largely to enhanced intermolecular interactions between the zwitterion and nearby serine molecules. This is also consistent with our free-energy sampling results that reveal significantly reduced free-energy barriers, thus, confirming the kinetic favorability for proton transfer. Our work illuminates key dynamic and kinetic features, including configurational and conformational entropy contributions from *ab initio* free-energy samplings that may largely govern the CO_2_ chemisorption process, which cannot be described by static quantum mechanical calculations. Our work can also aid in further studies of other potential AAILs and in designing optimal ionic liquid solvents for the post-combustion and direct air capture of the CO_2_ process.

## Supporting Information Available

Detailed computational methods, sensitivity on the level of theory (functional), metadynamics setup, convergence, and collective variable specifications (PDF)
