# Supplementary material for: Elucidating the Molecular Mechanism of CO2 Capture by Amino Acid Ionic Liquids
Source: J Am Chem Soc. 2023 Jul 13;145(29):15663–7. doi: 10.1021/jacs.3c03613 (PMC10375530; doi:10.1021/jacs.3c03613)
Supplement: Supplementary file 1 — ja3c03613_si_001.pdf [file ja3c03613_si_001.pdf]

Supporting Information for:

Elucidating the Molecular Mechanism of CO_2 Capture by Amino Acid Ionic Liquids

Bohak Yoon and Gregory A. Voth*

Department of Chemistry, Chicago Center for Theoretical Chemistry, The James Franck Institute, and Institute for Biophysical Dynamics, The University of Chicago, Chicago, IL 60637, USA

*Corresponding Author: gavoth@uchicago.edu

Number of Pages: 8

Number of Figures: 3

Number of Tables: 1

Detailed Description of Computational Methods and Simulation Setup

Ab initio molecular dynamics (AIMD) simulations based on density functional theory were performed with the CP2K software.[1] To prepare the AIMD simulations, all-atom classical molecular dynamics simulations were first computed using the LAMMPS MD software.[2] The simulation box containing 15 amino acid ionic liquids with cation and anion pairs of cholinium and serine with five CO_2 molecules was prepared using PACKMOL.[3] The cubic simulation box was run for 10 ns in the constant NPT ensemble, from which the equilibrated density is obtained. Thereafter, the simulation box was equilibrated under the NVT ensemble for 5 ns, followed by production runs for 15 ns. The final configuration from the production runs is used as the initial structure for AIMD runs. All AIMD simulations are computed at 300 K. The generalized gradient approximation (GGA) functional of revised Perdew, Berke, and Ernzerhof (revPBE) is used.[4, 5] The conclusions drawn from this work are unaffected by choice of functionals (see below). Norm-conserving Goedecker-Teter-Hutter (GTH) pseudopotentials are employed to describe the interactions between ionic cores and valence electrons.[6] A hybrid Gaussian and plane-waves (GPW) method[7] is used in the QUICKSTEP molecule[8], in which atom-centered Gaussian-type orbitals are used to describe the wave functions and an auxiliary plane wave basis set for expansion of the electron density. A triple-zeta Gaussian basis set with two polarization function sets (TZV2P) is employed; a plane-wave kinetic energy cutoff of 450 Ry is used. The Brillouin zone was sampled using only the gamma point. The semi-empirical dispersion corrections developed by Grimme (DFT-D3) with Becke-Johnson (BJ) damping is used to treat the long-range van der Waals interaction.[9] A timestep of 0.5 fs is used to integrate the equations of motion. AIMD simulations were run for 15 ps under the constant NVT ensemble, followed by production runs for 90 ps. Thereafter, well-tempered metadynamics simulations[10, 11] in conjunction with PLUMED plug-in[12] were carried out for the free-energy sampling of reaction pathways. More details on metadynamics simulation setup, convergence tests, and transition state verifications can be found below.

Sensitivity of Functional Choices (Levels)

All simulations are prepared with *ab initio* molecular dynamics (AIMD) augmented with well-tempered metadynamics. To check the sensitivity of the density functional theory (DFT) exchange-correlation functionals on our simulations and the corresponding results, we also performed additional free-energy sampling calculations for the two-step reaction pathways discussed in the main text. Here, the generalized gradient approximation (GGA) level of theory of the revised Perdew, Burke, Ernzerhof (revPBE) functional with is compared with that of Becke, Lee-Yang-Parr (BLYP) functional[13, 14], metaGGA level of theory of revised Tao, Perdew, Staroverov, Scuseria (revTPSS)[15, 16] and Strongly Constrained and Appropriately Normed (SCAN)[17] semi-local functionals. The corresponding Helmholtz free-energy barrier (under NVT ensemble) between the initial and the transition states for the two-step reaction pathways during CO_2 capture process are reported in Table S1. As illustrated in Table S1, the relative free-energy barrier (*FEB*) is reported for the reactions of case (a), (b), and (c), as described in the main text. The conclusions made from our simulation results on the *FEB* are unaffected by the higher level of DFT functionals (metaGGA), and revPBE (GGA) functional used in our simulations is suitable.

Table S1. Free energy barrier (*FEB*) for the reactions of case (a), (b), and (c) (i.e., FEB_a, FEB_b, FEB_c) based on various functionals of BLYP, revPBE, revTPSS, and SCAN with the level of theory of GGA and metaGGA from AIMD simulations for the two-step reaction pathways for CO_2 capture by amino acid ionic liquids (AAIL) studied in this work.

Functional	*Functional (theory) level*	FEB_a	FEB_b	FEB_c
BLYP	GGA	14.8 ± 0.3	9.5 ± 0.3	19.1 ± 0.4
revPBE	GGA	17.2 ± 0.2	12.1 ± 0.2	22.5 ± 0.3
revTPSS	metaGGA	17.8 ± 0.2	12.7 ± 0.3	23.0 ± 0.3
SCAN	metaGGA	17.5 ± 0.3	12.3 ± 0.2	22.7 ± 0.2

Metadynamics Simulations Details – I) Setup

Chemisorption of CO_2 by AAILs can be initiated from nucleophilic attack by a basic N atom in serine (anion) at an electrophilic C atom in CO_2, forming zwitterion. Thereafter, the zwitterion may undergo three possible routes involving proton transfer for its conversion into carbamate products. The overall reaction pathways are described in Figure 1 of the main text. Here, the three possible routes from the zwitterion intermediate include the followings: (a) intermolecular proton transfer to the O atom in serine, (b) intermolecular proton transfer to the N atom in serine, and (c) intramolecular proton transfer to the O atom within the zwitterion (as shown in Figure 1 of the main text). To investigate the thermodynamic and kinetic favorability of the reaction paths described herein, we first computed *ab initio* metadynamics. A representative simulation setup with 15 AAIL pairs of cholinium (cation) and serine (anion) with five CO_2 molecules in a cubic periodic box is illustrated in Figure 2 of the main text.

Metadynamics Simulations Details – II) Convergence

The free-energy barriers for the reaction of CO_2 by AAILs are calculated from AIMD with well-tempered metadynamics simulations. The three reaction cases of (a), (b), and (c) are the followings: (a) intermolecular proton transfer to the O atom in serine, (b) intermolecular proton transfer to the N atom in serine, and (c) intramolecular proton transfer to the O atom within the zwitterion (as shown in Figure 1 of the main text). As shown in Figure S1, the convergence of free-energy barriers is obtained after 20 (x 10^5) number of timestep for AIMD-metadynamics for all cases of (a), (b), and (c).

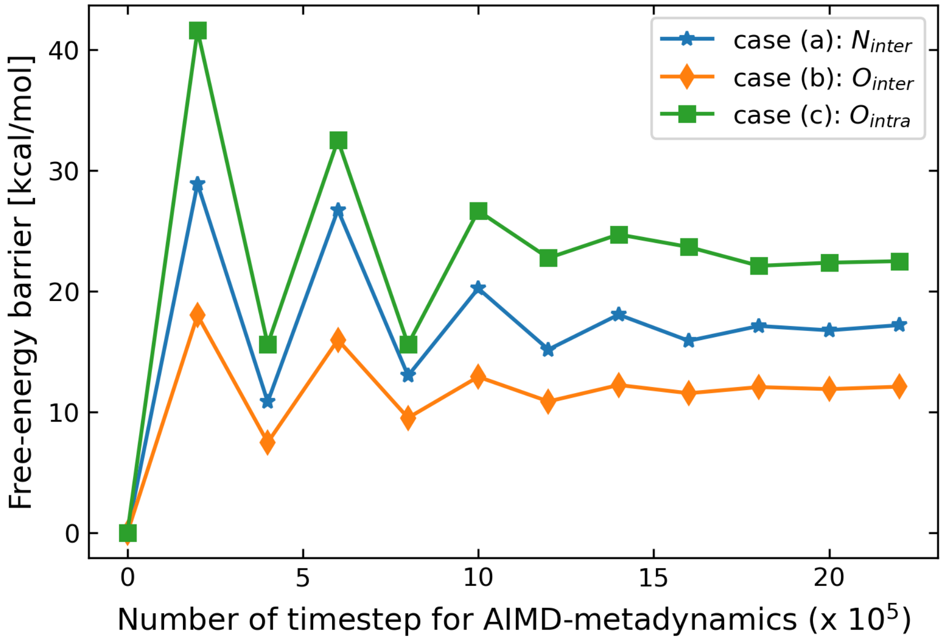

Figure S1. Free-energy barrier distributions during AIMD-metadynamics simulations for the two-step reaction pathways for CO_2 capture by amino acid ionic liquids (AAILs) studied in this work. The three reaction pathways involving proton transfer (a) intermolecularly to a nitrogen atom in serine (N_{inter}, in blue star), (b) intermolecularly to an oxygen atom in serine (O_{inter}, in orange diamond), and (c) intramolecularly to an oxygen atom within zwitterion (O_{intra}, in green square) are indicated.

Thereafter, another convergence is confirmed for the AIMD well-tempered metadynamics by investigating the deposited Gaussian hill heights. For the well-tempered algorithm, the Gaussian hill height should decay to zero once fully converged, which is also shown and confirmed with Figure S2 around 20 (x 10^5) number of timestep.

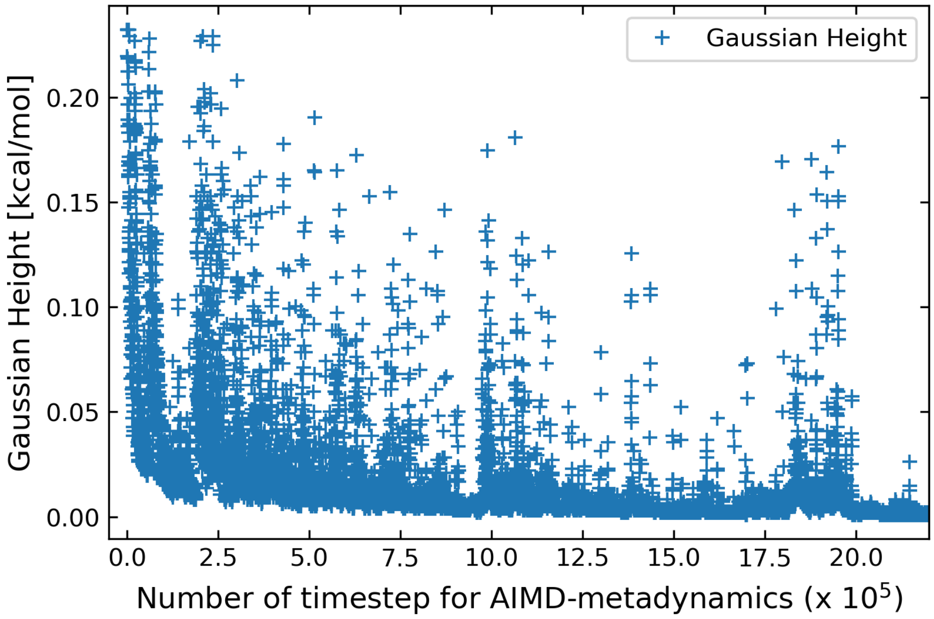

Figure S2. Gaussian hill height deposited distributions during AIMD-metadynamics with well-tempered algorithim for the two-step reaction pathways for CO_2 capture by amino acid ionic liquids (AAILs) studied in this work. The simulations are fully converged once Gaussian hill decays to zero.

Metadynamics Simulations Details – III) Transition state verifications

A large set of unbiased AIMD simulations were computed, in which at least 17 different molecular configurations near the identified transition state region during the CO_2 reactions with AAILs were simulated with random Boltzmann distributed velocities. It was subsequently determined, through these simulation runs, that the unbiased MD simulations fell into reactant or product states as represented along the chosen CV space, after a true reactive trajectory had been identified.

Metadynamics Simulations Details – IV) CV specifications

A free-energy surface (FES) was obtained from AIMD coupled with well-tempered metadynamics for the carbamate formation during CO_2 chemisorption process by AAILs. Here, the collective variables (CVs) chosen employ two collective variables. The first CV (ξ_3) is a bond distance between the N atom in serine molecule and the C atom in CO_2 molecule. A relatively larger value of ξ_3 indicates that the serine and CO_2 molecules are separated with no significant interaction between the two, while a comparatively smaller value of ξ_3 indicates that the zwitterion is formed. The second CV is composed of two coordination numbers (CNs): (i) a bond distance-dependent coordination number between a proton and the base N atom in zwitterion (ξ_1), and (ii) a bond distance-dependent coordination number between the proton and the base N atom in nearby serine molecule (ξ_2). Here, a linear combination of the two CVs (ξ_1–ξ_2) can conveniently describe

protonation states of both the zwitterion and nearby serine molecule. A positive value (specifically a value of unity, 1, due to ξ_1–ξ_2 = 1–0 = 0) indicates that the proton is fully bound to the zwitterion, while there is no significant interaction between the proton and serine molecule; a negative value (specifically a value of -1, due to ξ_1–ξ_2 = 1–0 = 0) denotes that the proton is successfully transferred from the zwitterion to the nearby serine. The CNs were defined using a rational switching function for the purpose of ensuring differentiability and the values of the CVs were set to unity or negative unity. The switching function description of the second CV employing two CNs are the followings.

$$\xi_1 = CN_{N,zwit-H} = \begin{cases} 1 & if, r_{ab} \leq 0 \\ \sum_{a \in N,zwit, b \in H} \dfrac{1 - \left(\frac{r_{ab}}{r_0}\right)^n}{1 - \left(\frac{r_{ab}}{r_0}\right)^m} & if, r_{ab} > 0 \end{cases}$$

$$\xi_2 = CN_{N,ser-H} = \begin{cases} 1 & if, r_{ab} \leq 0 \\ \sum_{a \in N,ser, b \in H} \dfrac{1 - \left(\frac{r_{ab}}{r_0}\right)^n}{1 - \left(\frac{r_{ab}}{r_0}\right)^m} & if, r_{ab} > 0 \end{cases}$$

$$-1 \leq \xi_1 - \xi_2 (= CN_{N,zwit-H} - CN_{N,ser-H}) \leq 1$$

The second CV (CV2) employing two CNs guarantee the followings: if the proton is bound to zwitterion, $CV_2 = \xi_1 - \xi_2 = 1 - 0 = 1$; if the proton is bound to the serine molecule, $CV_2 = \xi_1 - \xi_2 = 0 - 1 = -1$. The distribution of CVs are illustrated in Figure S3, also confirming the convergence of AIMD well-tempered metadynamics.

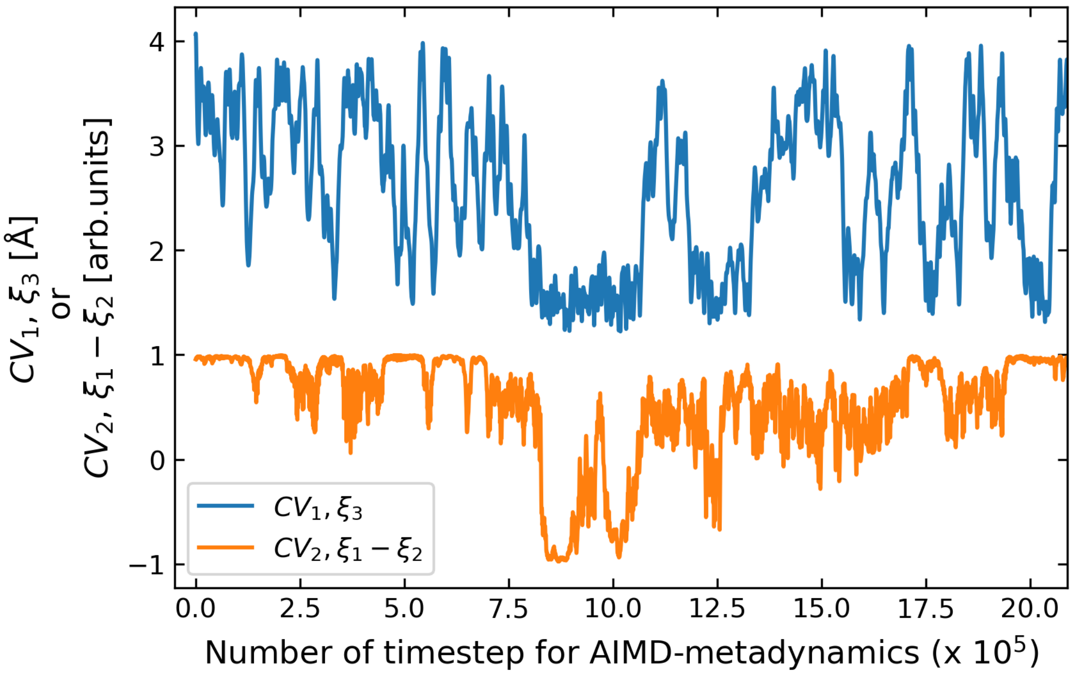

Figure S3. Collective variables (CVs) distributions during the AIMD-metadynamics with well-tempered algorithm. CV_1 is ξ_3, mimicking the bond distance between N and C atoms, while CV_2 is ξ_1–ξ_2, denoting a difference between two coordination numbers for protonation states.

REFERENCES

(1) Kuhne, T. D.; Iannuzzi, M.; Del Ben, M.; Rybkin, V. V.; Seewald, P.; Stein, F.; Laino, T.; Khaliullin, R. Z.; Schutt, O.; Schiffmann, F.; et al. CP2K: An electronic structure and molecular dynamics software package - Quickstep: Efficient and accurate electronic structure calculations. *J Chem Phys* **2020**, *152* (19), 194103. DOI: 10.1063/5.0007045.

(2) Thompson, A. P.; Aktulga, H. M.; Berger, R.; Bolintineanu, D. S.; Brown, W. M.; Crozier, P. S.; in 't Veld, P. J.; Kohlmeyer, A.; Moore, S. G.; Nguyen, T. D.; et al. LAMMPS - a flexible simulation tool for particle-based materials modeling at the atomic, meso, and continuum scales. *Computer Physics Communications* **2022**, *271*, 108171. DOI: 10.1016/j.cpc.2021.108171.

(3) Martinez, L.; Andrade, R.; Birgin, E. G.; Martinez, J. M. PACKMOL: a package for building initial configurations for molecular dynamics simulations. *J Comput Chem* **2009**, *30* (13), 2157-2164. DOI: 10.1002/jcc.21224.

(4) Perdew, J. P.; Burke, K.; Ernzerhof, M. Generalized Gradient Approximation Made Simple. *Phys Rev Lett* **1996**, *77* (18), 3865-3868. DOI: 10.1103/PhysRevLett.77.3865.

(5) Zhang, Y.; Yang, W. Comment on "Generalized Gradient Approximation Made Simple". *Physical Review Letters* **1998**, *80* (4), 890-890. DOI: 10.1103/PhysRevLett.80.890.

(6) Goedecker, S.; Teter, M.; Hutter, J. Separable dual-space Gaussian pseudopotentials. *Phys Rev B Condens Matter* **1996**, *54* (3), 1703-1710. DOI: 10.1103/physrevb.54.1703.

(7) Wilhelm, J.; Hutter, J. Periodic GW calculations in the Gaussian and plane-waves scheme. *Physical Review B* **2017**, *95* (23). DOI: 10.1103/PhysRevB.95.235123.

(8) VandeVondele, J.; Krack, M.; Mohamed, F.; Parrinello, M.; Chassaing, T.; Hutter, J. Quickstep: Fast and accurate density functional calculations using a mixed Gaussian and plane waves approach. *Computer Physics Communications* **2005**, *167* (2), 103-128. DOI: 10.1016/j.cpc.2004.12.014.

(9) Smith, D. G.; Burns, L. A.; Patkowski, K.; Sherrill, C. D. Revised Damping Parameters for the D3 Dispersion Correction to Density Functional Theory. *J Phys Chem Lett* **2016**, *7* (12), 2197-2203. DOI: 10.1021/acs.jpclett.6b00780.

(10) Barducci, A.; Bussi, G.; Parrinello, M. Well-tempered metadynamics: a smoothly converging and tunable free-energy method. *Phys Rev Lett* **2008**, *100* (2), 020603. DOI: 10.1103/PhysRevLett.100.020603.

(11) Laio, A.; Parrinello, M. Escaping free-energy minima. *Proc Natl Acad Sci U S A* **2002**, *99* (20), 12562-12566. DOI: 10.1073/pnas.202427399.

(12) Bonomi, M.; Branduardi, D.; Bussi, G.; Camilloni, C.; Provasi, D.; Raiteri, P.; Donadio, D.; Marinelli, F.; Pietrucci, F.; Broglia, R. A.; et al. PLUMED: A portable plugin for free-energy calculations with molecular dynamics. *Computer Physics Communications* **2009**, *180* (10), 1961-1972. DOI: 10.1016/j.cpc.2009.05.011.

(13) Becke, A. D. Density-functional exchange-energy approximation with correct asymptotic behavior. *Physical Review A* **1988**, *38* (6), 3098-3100. DOI: 10.1103/physreva.38.3098.

(14) Lee, C.; Yang, W.; Parr, R. G. Development of the Colle-Salvetti correlation-energy formula into a functional of the electron density. *Physical Review B* **1988**, *37* (2), 785-789. DOI: 10.1103/physrevb.37.785.

(15) Perdew, J. P.; Ruzsinszky, A.; Csonka, G. I.; Constantin, L. A.; Sun, J. Workhorse Semilocal Density Functional for Condensed Matter Physics and Quantum Chemistry. *Physical Review Letters* **2009**, *103* (2). DOI: 10.1103/physrevlett.103.026403.

(16) Tao, J.; Perdew, J. P.; Staroverov, V. N.; Scuseria, G. E. Climbing the Density Functional Ladder: Nonempirical Meta–Generalized Gradient Approximation Designed for Molecules and Solids. *Physical Review Letters* **2003**, *91* (14). DOI: 10.1103/physrevlett.91.146401.

(17) Sun, J.; Ruzsinszky, A.; Perdew, J. P. Strongly Constrained and Appropriately Normed Semilocal Density Functional. *Physical Review Letters* **2015**, *115* (3). DOI: 10.1103/physrevlett.115.036402.
